# Sex- and Age-specific Outcomes After Ruptured Abdominal Aortic Aneurysm Repair: A Nationwide Cohort Study

**DOI:** 10.1097/SLA.0000000000007191

**Published:** 2026-08-14

**Authors:** Mathijs J. Biemond, Jan van Schaik, Jaap F. Hamming, Jorg L. de Bruin, Jeroen J.W.M. Brouwers, Mark Dirven, Gabor Gäbel, Joost A. van Herwaarden, Vincent Jongkind, Jon S. Matsumura, Barend M.E. Mees, Abbey Schepers, Jean-Paul P.M. de Vries, Joost R. van der Vorst, Jan H.N. Lindeman

**Affiliations:** 1 Department of Vascular Surgery, Leiden University Medical Center, Leiden, the Netherlands; 2 Department of Vascular Surgery, Erasmus Medical Center, Rotterdam, the Netherlands; 3 Department of Vascular Surgery, Radboud University Medical Center, Nijmegen, the Netherlands; 4 Department of Vascular Surgery, Evangelisches Krankenhaus Herne, Herne, Germany; 5 Department of Vascular Surgery, University Medical Center Utrecht, Utrecht, the Netherlands; 6 Department of Vascular Surgery, Amsterdam University Medical Center, Amsterdam, the Netherlands; 7 Department of Surgery, University of Colorado School of Medicine, Aurora, CO; 8 Department of Vascular Surgery, Maastricht University Medical Center, Maastricht, the Netherlands; 9 Department of Vascular Surgery, University Medical Center Groningen, Groningen, the Netherlands

**Keywords:** ruptured abdominal aortic aneurysm, nationwide cohort, sex differences, endovascular and open repair, long-term survival, postdischarge care

## Abstract

**Background:**

Ruptured abdominal aortic aneurysm (RAAA) remains a medical catastrophe. Although early outcomes improved, contemporary data integrating early and long-term survival, and postdischarge care needs across sex- and age groups is limited.

**Methods:**

This nationwide cohort included Dutch RAAA repairs between 2014 and 2023. Dutch Surgical Aneurysm Audit data were linked to Statistics Netherlands registries. Outcomes included 90-day mortality, long-term survival, and postdischarge care.

**Results:**

Of the 3642 patients, 15.2% were women, who were older (76.3 vs. 74.0 y) and more often required open repair (61.4% vs. 54.0%). Ninety-day mortality increased with age and was higher in women (39.1% vs. 29.1%), but female sex was not independently associated with early mortality after adjustment.

Among 90-day survivors, absolute long-term survival was similar, but relative survival was lower in women (5-year relative survival 68.0% vs. 78.3%). Women were less often discharged home (42.4% vs. 56.7%) and more frequently required early homecare (47.7% vs. 34.7%). Reinterventions were more common after EVAR than open repair and occurred less frequently in women.

**Conclusion:**

In this nationwide linked audit-registry analysis, outcomes after RAAA repair improved substantially, despite lower estimated in-hospital turndown rates than in earlier eras. The higher early mortality in women WAS reflected by differences in age, presentation severity, and repair type – possibly reflecting less favorable aneurysm anatomy. Female sex also associated with an increased long-term excess mortality and greater postdischarge dependency. These findings indicate that optimization of care for women with a RAAA is not confined to perioperative mortality, but extends across the entire care pathway.

A ruptured abdominal aortic aneurysm (RAAA) is a life-threatening emergency, with reported mortality rates approaching 80%.^1,2^ Over the past 2 decades, RAAA management and epidemiology have changed considerably, with increased use of endovascular aneurysm repair (EVAR) and improved care trajectories on the one hand, and an aging patient population on the other. These improvements resulted in faster recovery and possibly improved short-term survival.^3–5^ While the improvements in early surgical outcomes are well documented,^3^ contemporary long-term outcome data is limited.

In addition, while long-term outcome research has a traditional focus on overall survival, possible sex-related differences^6^ in outcomes and secondary outcome measures such as reinterventions received limited attention. While older adults value life expectancy, they also consider functional independence a critical determinant of quality of life.^7^ This aspect and the strains functional dependence puts on health care systems^8^ are so far largely ignored.

This study evaluates contemporary short-term, postdischarge, and long-term outcomes following RAAA repair in the Netherlands during 2014–2023, focusing on sex- and age-specific differences in presentation, treatment, and outcomes, with specific reference to national trends in ruptured AAA management over the last 3 decades.

## METHODS

### Study Design

This retrospective cohort study utilized anonymized data from the Dutch Surgical Aneurysm Audit (DSAA) and linked national registries accessible through Statistics Netherlands.^9^ The scientific board of the DSAA and the Leiden University Medical Center review board approved the study (No. 2025-054). Under Dutch law no informed consent was required due to the use of anonymized data. This study adheres to the Strengthening the Reporting of Observational Studies in Epidemiology (STROBE) guidelines.^10^

### Study Setting

The study was primarily based on DSAA-recorded patients undergoing primary RAAA repair in the Netherlands between January 1st 2014, and December 31st 2023. The DSAA, a mandatory nationwide clinical registry for aneurysm repairs since 2013, provides detailed patient characteristics and procedural information with high completeness. DSAA data were linked to Statistics Netherlands registries,^9^ providing full longitudinal information on hospitalizations, mortality, medication use, and homecare. In addition, Statistics Netherlands hospital discharge and mortality data was used to derive population-level counts of RAAA admissions, repairs, and RAAA-related hospital deaths between 1995 and 2023 to contextualize time trends.

### Data Sources

Procedure-specific details were extracted from the DSAA. Admission and discharge information, including origin and destination after discharge, as well as information on readmissions (using ICD-10 codes) were retrieved from the Statistics Netherlands Hospital Discharge Registry. Dates and causes of death up to December 2024 were obtained from the Mortality Registry. Baseline medication use was derived from the Medication Registry, and homecare utilization (available from January 2015) from the Homecare Registry. Registries from Statistics Netherlands include cross-database unique personal and admission identifiers for linkage. The same personal identifier was assigned to DSAA cases using deterministic matching using sex, date of birth, and postal code (95.2% successfully matched). For contextual analyses of national RAAA trends (1995–2023), the Statistics Netherlands registries were additionally queried for RAAA admissions, repairs, and RAAA-related hospital deaths, defined using cause-of-death coding. Coding definitions are provided in Supplemental Table 1, Supplemental Digital Content 1, https://links.lww.com/SLA/F926.

### Participants

All patients undergoing primary RAAA between 2014 and 2023 were included. In the DSAA, RAAA is manually recorded by the operating surgeon after repair. Patients with a thoracoabdominal or isolated iliac aneurysm, and those lacking a unique identifier required for registry linkage were excluded.

### Variables

Outcomes for the DSAA cohort encompassed type of repair, survival, and postdischarge health care utilization (destination after discharge, homecare use, hospital readmissions, and aortic reinterventions). Survival outcomes included 90-day all-cause mortality, and long-term overall and relative survival at 1, 3, and 5 years among 90-day survivors. Hospital readmissions were defined as inpatient admission within 90 days after discharge from the index RAAA repair. Aortic reinterventions were defined as aorta- or graft-related procedures for complications of the index RAAA repair registered in DSAA data.

### Statistical Methods

Data were analyzed using R (version 4.4.1) and RStudio (version 2024.09.0). Differences in baseline characteristics were assessed using *t* test, Mann-Whitney *U* test, or χ^2^ tests.

Ninety-day all-cause mortality was stratified by sex, age, and repair type (EVAR vs. OR). Multivariable logistic regression evaluated associations with 90-day mortality; covariates included sex, age, repair type, presentation severity, and comorbidity. Kaplan-Meier curves visualized long-term survival, and relative survival among 90-day survivors was estimated at 1, 3, and 5 years using the Ederer-II method with age-, sex-, and period-specific lifetables from Statistics Netherlands. Homecare data (visualized using Alluvial diagrams) were available from 2015; proportions were normalized across years to ensure consistent visualization. Reintervention incidences were assessed using Aalen-Johansen plots and Fine-Gray models to account for competing mortality. For contextual analyses, Statistics Netherlands data described trends in RAAA admissions, repairs, and RAAA-related hospital deaths in 2 time periods. The number of nonoperated RAAA cases was estimated by subtracting in-hospital deaths following repair from total in-hospital RAAA deaths, to estimate turndown rates over time. *P* values below 0.05 were considered statistically significant.

## RESULTS

### National Trends in RAAA Management

Statistics Netherlands data show a marked shift in RAAA management over time. Between 1995 and 2004, an estimated 70.6% of the admitted RAAA patients did not receive operative repair, compared with 29.7% in the contemporary period (2014–2023). Temporal improvements in short-term mortality following repair are illustrated in Supplemental Figure 1, Supplemental Digital Content 1, https://links.lww.com/SLA/F926. Compared with 1995–2004, 90-day mortality decreased from 45.0% to 29.1% in men and from 56.0% to 39.1% in women.

### Contemporary Cohort

Supplementary Table 9, Supplemental Digital Content 1, https://links.lww.com/SLA/F972 summarizes the baseline characteristics for the 3642 patients undergoing RAAA repair from 2014 to 2023, an interval characterized by further implementation of EVAR (Supplemental Fig. 2, Supplemental Digital Content 1, https://links.lww.com/SLA/F926). 15.2% were women, who were older at repair (mean age 76.3 y vs. 74.0 y in men, *P*<0.001), had smaller aneurysm diameters (median 70.6 mm vs. 80.0 mm, *P*<0.001), and were more likely to receive OR (61.4% vs. 54.0% in men, *P*=0.002). Patients receiving OR were younger, more often had complex aneurysm anatomy (juxtarenal or suprarenal), and required a longer hospital stay (Supplemental Table 2, Supplemental Digital Content 1, https://links.lww.com/SLA/F926).

### Short-term Mortality

Ninety-day mortality risk increased linearly with age for both sexes and repair types (*P*<0.001; Fig. 1 and Supplemental Table 3, Supplemental Digital Content 1, https://links.lww.com/SLA/F926). Among men, mortality with EVAR rose from approximately 9% for those under 60 years to 35% for those aged >86, and from 14% to over 50% for OR for the same age range. Among women, EVAR mortality increased from 25% to 40%, and OR mortality from 22% to 70%.

**FIGURE 1:**
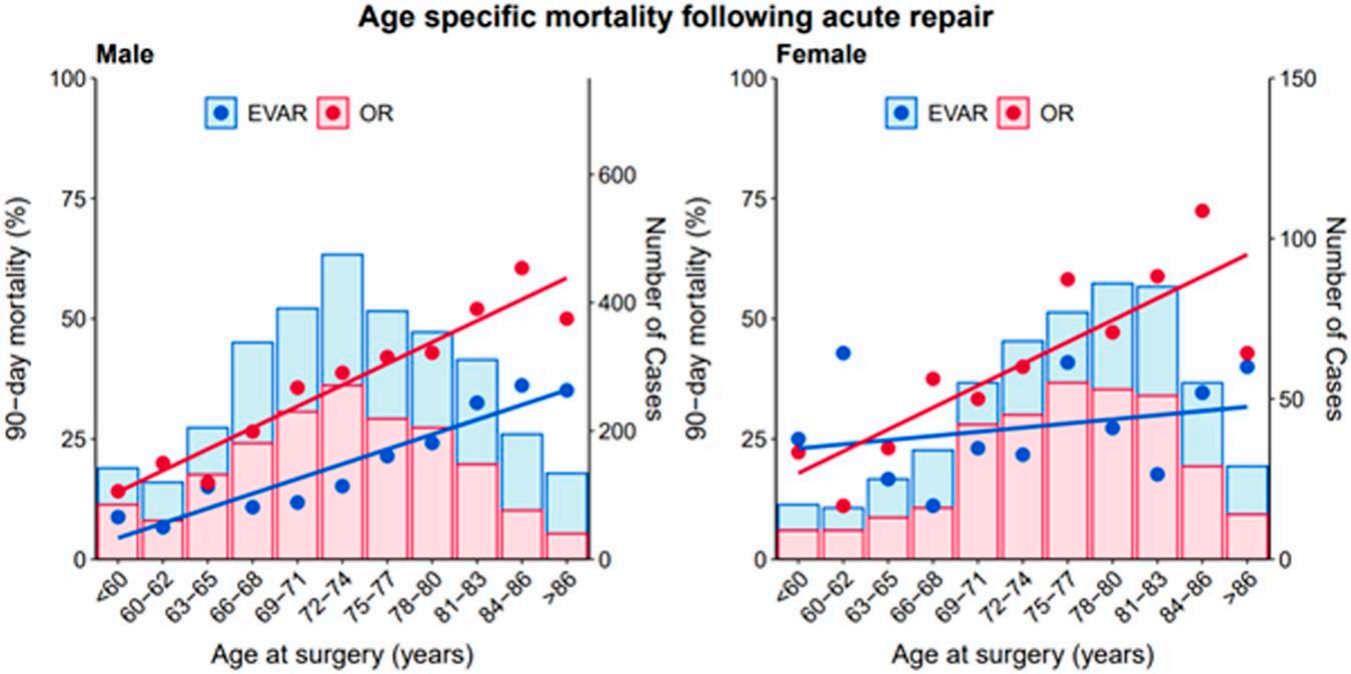
Age distribution (bars) at- and age-specific all-cause mortality (points) following RAAA repair

Female sex was independently associated with higher 90-day mortality in a regression model including sex, age, and repair type (OR 1.29, 95% CI: 1.05–1.57; Supplemental Table 4, Supplemental Digital Content 1, https://links.lww.com/SLA/F926). Significance was lost after additional adjustment for presentation severity (Hb, systolic blood pressure, Glasgow Coma Scale, aneurysm diameter) and comorbidity (OR 1.13, 95% CI: 0.87–1.45; Supplemental Table 5, Supplemental Digital Content 1, https://links.lww.com/SLA/F926). Similarly, no sex differences were observed in a sensitivity analysis restricted to infrarenal aneurysms (OR 0.96, 95% CI: 0.69-1.33; Supplemental Table 6, Supplemental Digital Content 1, https://links.lww.com/SLA/F926).

### Long-term Survival

Kaplan-Meier curves (conditional on 90-day survival) demonstrated similar survival between men and women up to 5 years after repair (Supplemental Fig. 3, Supplemental Digital Content 1, https://links.lww.com/SLA/F926). Nevertheless, relative survival analysis indicated a compromised long-term survival compared with the general Dutch population, with a more outspoken deficit in women (Supplementary Fig. 7, Supplemental Digital Content 1, https://links.lww.com/SLA/F972). At 5 years, survival for RAAA patients over 75 years was 52.0% for men and 49.8% for women, compared with 66.5% and 73.2% in the age- and sex-matched Dutch population, with relative survival of 78.3% and 68.0% respectively (Supplemental Table 7, Supplemental Digital Content 1, https://links.lww.com/SLA/F926).

Overall survival (ie, including early mortality) stratified by repair type and sex confirmed the early mortality disadvantage for OR and women, with subsequent convergence of survival probabilities between EVAR and OR over time (Fig. 2).

**FIGURE 2:**
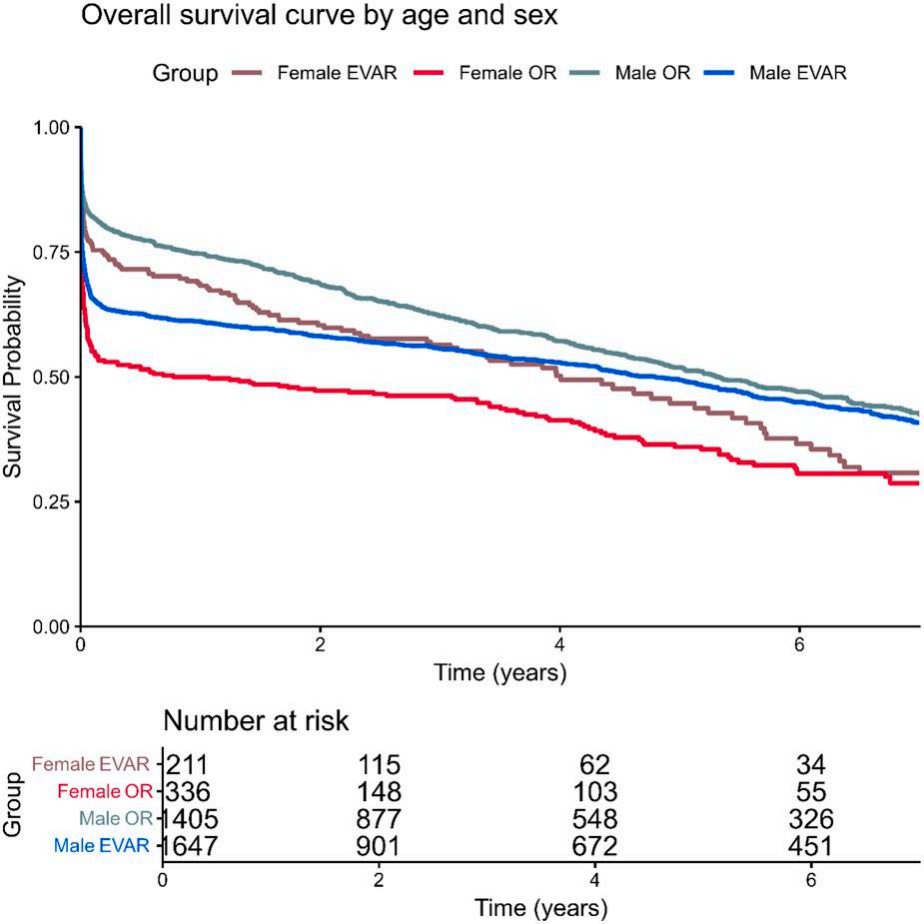
Kaplan-Meier survival curve for overall survival by sex and repair type.

### Postdischarge Trajectory

Alluvial diagrams in (Supplementary Fig. 8, Supplemental Digital Content 1, https://links.lww.com/SLA/F972) summarize discharge and postdischarge care. The diagrams highlight pronounced sex-specific variations in care utilization. Among men, 56.7% returned home postadmission, compared with 42.4% of women, a disparity attributable to the higher in-hospital mortality rate in women and to higher incidences of nursing home and rehabilitation center admissions for women. For patients aged over 75 years, a notably higher proportion experienced in-hospital death, or transition to nursing home or rehabilitation care. Early (within 90 d postrepair) homecare utilization shows that men received less homecare (34.7%) than women (47.7%).

One-year postdischarge, nearly half of men (49%), but only one third of the women (33%) reside at home without homecare. However, profound variations are observed across age, sex, and repair type (Supplemental Figs. 4–6, Supplemental Digital Content 1, https://links.lww.com/SLA/F926).

### Readmissions

Early (within 90-day postdischarge) readmission rates and patterns were similar across all sex and age groups (16.0%–17.7%, *P*=0.824; Supplemental Table 8, Supplemental Digital Content 1, https://links.lww.com/SLA/F926). Cardiovascular causes (ICD-10 “I-codes” excluding aneurysm-related codes) dominated readmissions (1.7%–21.5%), followed by surgical complications (13.1%–20.9%). The remainder of resubmissions related to highly heterogenous indications.

### Aortic Reinterventions

Late aortic reintervention incidences were higher after EVAR, accumulating to ∼12% for males and 10% for females at 7 years (Supplemental Fig. 7, Supplemental Digital Content 1, https://links.lww.com/SLA/F926). Males had a higher reintervention hazard than females (subdistribution hazard ratio (sHR) 1.84, 95% CI: 1.11–3.08, *P*=0.019) in a competing risk model adjusted for sex and repair type, and the reintervention rate after EVAR exceeded that of OR (sHR 4.29, 95% CI: 3.10–5.95, *P*<0.001).

## DISCUSSION

This study provides a comprehensive evaluation of contemporary RAAA care across the entire care pathway by linking detailed surgical audit data to longitudinal national registries. Outcomes after RAAA repair in the Netherlands have improved substantially over the last 3 decades, with lower short-term mortality and a markedly reduced estimated in-hospital turndown rate. Within the contemporary cohort, pronounced sex differences persisted in presentation, treatment, and outcomes. Women presented at older age and were more frequently treated with OR, factors that likely contribute to their higher 90-day mortality. Importantly, this early excess mortality was largely explained by differences in age, presentation severity, repair type, and anatomic distribution. Beyond the early postoperative period, women continued to demonstrate more pronounced long-term excess mortality after successful repair and greater postdischarge health care needs. Reintervention rates were higher after EVAR but were lower among women. These findings indicate that the female disadvantage after RAAA is not confined to perioperative mortality, but extends across the entire care pathway.

This study relied on 90-day rather than 30-day mortality for short-term outcomes, as this better captures delayed complications and provides a more accurate prediction of long-term outcomes.^11,12^ Consistent with Swedish registry data,^13^ short-term survival after RAAA repair has improved over time, with contemporary outcomes outperforming historical benchmarks^14^ (Supplemental Fig. 1, Supplemental Digital Content 1, https://links.lww.com/SLA/F926). These improvements likely reflect a combination of advances in perioperative care and widespread adoption of EVAR.^15^ Superior short-term outcomes for EVAR align with recent registry and administrative-data studies,^16^ but contrast with reported outcome equivalence in randomized trials such as AJAX^17^ and IMPROVE,^18^ likely due to the eligibility criteria of these trials. In this population-based study, EVAR was essentially restricted to infrarenal aneurysms, whereas OR encompassed a broader anatomic spectrum, including a higher proportion of juxta- and suprarenal aneurysms, particularly in women. These case-mix differences likely contribute to the survival disadvantage observed for OR.

Several nonexclusive explanations may account for the persistent early survival disadvantage in women. First, RAAA in women presents at an older age, which shifts a larger proportion into higher risk strata, as age strongly correlates with perioperative mortality in this study and both in cohorts of elective and ruptured aneurysms.^19^ Second, women were more likely to receive OR with the accompanying higher procedural risk.^20^ This partly reflects the higher proportion of juxta- and suprarenal aneurysms – anatomical configurations that typically require open surgery – and may also relate to the observation that women less often meet EVAR device instruction for use (IFU) criteria (eg, shorter necks, greater angulation, and smaller iliac diameters).^19,21^ Finally, lower preoperative Hb and GCS values suggest a more compromised condition. While other studies reported higher perioperative mortality in women after both elective and ruptured AAA repair, even after adjustment for age and comorbidities,^6,22,23^ significance was lost after accounting for differences in age, presentation severity, repair type, and anatomic distribution. This implies that these factors – rather than a sex-related vulnerability – mediate the higher early mortality in women. It cannot be excluded that unmeasured elements like referral delays FURTHER contribute to the early survival disadvantage for women.

Absolute long-term survival (conditional on 90-day survival) was similar for men and women. Relative survival analysis, demonstrated loss of the usual female survival advantage, and as a consequence a more outspoken deficit compared with the general population. A comparable deficit was described for elective AAA repair,^24^ and the present findings extend this observation to RAAA repair. Unconditional (overall) survival analyses demonstrate a gradual convergence of survival outcomes for EVAR and OR over time. This may reflect survivorship bias, where higher perioperative mortality with OR leaves a more resilient cohort with more favorable long-term outcomes, while EVAR’s initial survival advantage is tempered by subsequent deaths among patients with greater frailty who could survive endovascular repair.^25,26^

Autonomy and independence are key quality of life (QoL) outcomes for older patients and represent key metrics for evaluating treatment efficacy in RAAA survivors, particularly among older adults, where comorbidities and functional recovery may outweigh technical success.^27^ Management decisions between repair and palliation inherently involve balancing survival benefit against expected postoperative QoL.^28^ Assessing outcomes such as the likelihood of regaining independence may therefore provide valuable context for these decisions. Postdischarge health care utilization, including homecare needs and institutional placements, is commonly used as a surrogate for functional recovery in administrative data.^29^ In this cohort, women (particularly older women) had lower rates of independent home return and greater reliance on formal homecare. While this pattern has been described in elective AAA,^30^ and aligns with the limited data for RAAA survivors,^31,32^ it does not necessarily indicate poorer clinical recovery in women. Rather, formal care use is influenced by broader demographic and social factors: older women are more often widowed or living alone, and may have fewer informal caregivers, whereas older men are reported to underutilize formal homecare services despite comparable needs.^33–35^ Thus, postdischarge dependency should be interpreted in this broader demographic context. Evidence from broader surgical populations suggests that structured frailty assessment during hospitalization, early mobilization, and multidisciplinary discharge planning are associated with better postoperative outcomes and increased home discharge rates.^36^ Such strategies may help reduce postdischarge dependency.

Despite ongoing concerns about device suitability in women,^19^ fewer reinterventions were observed among women in this cohort. Similarly, no significant sex difference was observed in a large US registry (HR ∼1.08 for women vs. men).^37^ By contrast, institutional series of elective EVAR performed outside device instructions for use report higher reintervention rates in women.^38,39^ However, these studies typically rely on Kaplan-Meier methods, that do not account for the higher competing mortality among women, thereby potentially overestimating the reintervention risk. Present findings, adjusted for competing mortality, suggest superior reintervention outcomes in women after EVAR, challenging the assumption that devices inherently favor male anatomy.

This study has limitations inherent to retrospective registry analyses. The observational design precludes causal inference; and selection and indication bias are likely, as treatment allocation and observed sex differences are potentially influenced by anatomy, hemodynamic status, and clinical decision-making not captured in this registry. In addition, exact treatment turndown data were unavailable. Therefore, treatment rates were approximated using national hospital discharge and mortality data and should be interpreted as population-level proxies, as they do not capture prehospital deaths or reasons for palliation. Data completeness varied, with homecare data being available only from 2015. Reinterventions were captured through DSAA entries, risking under capture of events outside the registry. The absence of direct quality of life and frailty measures limits exploration of unmeasured confounders affecting postrepair dependency and patients-centered outcomes. However, the Dutch health care system is characterized by equal access, standardized care pathways, and short transfer times, which likely mitigates some biases related to access and treatment standardization.

## CONCLUSION

This nationwide linked-data analysis demonstrates substantial improvements in outcomes after RAAA repair over the past 3 decades, alongside a markedly lower in-hospital turndown rate. Despite these advances, clinically relevant sex disparities persist. Women experience higher early mortality, greater long-term excess mortality, and a higher burden of postdischarge dependency. The excess early mortality in women was largely explained by differences in age, presentation, and treatment allocation, possibly reflecting less favorable aneurysm anatomy. In contrast, the mechanisms underlying the observed long-term excess mortality and postdischarge dependency remain incompletely understood. These findings indicate that the female disadvantage after RAAA and the need for optimization of care is not confined to perioperative mortality, but extends across the entire care pathway, from presentation and treatment selection to postdischarge recovery.

### Data Availability

The data underlying this study are not publicly available due to legal restrictions associated with Dutch privacy legislation and the terms of access to Statistics Netherlands (CBS) microdata. CBS microdata can only be accessed through a secured remote environment after formal application and approval. Researchers may request access to the underlying anonymized datasets through Statistics Netherlands (https://www.cbs.nl) and the Dutch Surgical Aneurysm Audit (DSAA), subject to the respective review and access procedures. The authors are not permitted to share the raw data.

## Supplementary Material

## DISCUSSANTS

### Gilbert R. Upchurch, Jr. (Gainesville, United States)

Thank you very much for your presentation. Thank you to the European Surgical Association for the privilege to speak from the floor. Professor Lindeman and his group in the Netherlands have really given us some of the best AAA clinical series reported, and this paper is a continuation. I have 2 questions. First, you had a significant decline in the number of people who presented with ruptured AAAs over time in your series. Do you think this is related to screening programs, or do you think this is related to fewer people smoking? It seems like both of those things may have contributed to this sharp decline in the number of patients presenting with ruptured AAAs.

My second question has to do with this concept of frailty, and I know you examined the Charlson Comorbidity Index in this series, but in the United States, we believe that frailty is really a risk factor for poor outcomes following any major surgery. I wondered whether, as you were pointing out these differences between males and females, you considered reviewing the CT scans on these patients to examine something as simple as psoas size as a measure of frailty in these patients?

### Response From Mathijs J. Biemond (Leiden, The Netherlands)

Thank you for your questions. Regarding the decline in ruptures, in the earlier period (1995–2004), we treated about 5,000 ruptures in 10 years with a turndown rate of 70%, meaning the true presentation of patients with a rupture would have been about 15,000 or 16,000. In the contemporary era (2014–2023), we only treated 3,500 patients, with a turndown rate of 30%, which means the total presentation is about 5,000 patients. An almost threefold fall. The Netherlands has never had a dedicated screening program, so it’s unlikely to reflect direct screening. However, in the same time period, the marked increase in CT, ultrasound, and MRI imaging has probably contributed to earlier detection. (This is also reflected by the increase of elective repairs). Reduced smoking is also a likely factor. Smoking effects take some time until it leads to the aneurysm. But in the 1950s, 1960s, about 90% of the Dutch males smoked compared to about 12% today, which very likely contributes to lower aneurysm prevalence in our population. Then the second question on frailty in women. Remarkably the Charlson Comorbidity Index (CCI) was comparable between sexes, which is remarkable as our women were 2 to 3 years older at time of repair. So, the CCI does not directly indicate a frailty difference. However, the women who were treated, more often received homecare before rupture, which could indicate greater frailty. However, this might also reflect demographic differences, as women are more likely to lack an informal caregiver than men.

### Bas Wijnhoven (Rotterdam, The Netherlands)

Thank you very much. I really want to congratulate you on this wonderful study. I think it’s very important to show the complete care pathway from diagnosis all the way to treatment and post-treatment. I just have a question: The database that you were using is a national surgical audit. I was wondering, how sure are you about the validity of this database? Has there been any research done to evaluate the completeness of the data? In other words, are all patients registered in the database? Because you said surgeons are registering their own patients, one might wonder whether they may exclude some patients that had mortality early on, et cetera. Can you assure us about the validity of the database that was used for this study? Thank you.

### Response From Mathijs J. Biemond (Leiden, The Netherlands)

There is no published formal validation study of the DSAAA, although random in-hospitals checks of data accuracy is performed. Secondly, we were also able to assess validity ourselves using our two linked sources: Dutch Surgical Aneurysm Audit, completed in detail by operating surgeons, and the less detailed Statistics Netherlands data, which captures the same ruptures. Comparing the number of admitted ruptures and elective repairs between the two sources showed close agreement. So, we performed a cross-reference ourselves to check the validity of the data set, although this has not been published.

### John Reynolds (Dublin, Ireland)

In terms of the implications of these data, and the sex differential in outcomes, how do you intend to use these findings nationally, in terms of targeted screening programs, public awareness, and so on?

### Response From Mathijs J. Biemond (Leiden, The Netherlands)

Thank you for your question. I think public screening might not be required, but two things are very interesting. First, women presented in a more compromised physiological state, raising the possibility that aneurysm ruptures are recognized less promptly in women, perhaps akin to myocardial infarction, which presents differently in women. This warrants further study. Second, women received open repair more often, partly related to the fact that they had more suprarenal and juxtarenal aneurysms. But revisiting the preoperative CT imaging to assess whether EVAR would have been feasible in some of those cases could help improve their outcomes.
