# Supplementary material for: Sex- and Age-specific Outcomes After Ruptured Abdominal Aortic Aneurysm Repair: A Nationwide Cohort Study
Source: Ann Surg. 2026 Aug 14;284(5):943–9. doi: 10.1097/SLA.0000000000007191 (PMC13652115; doi:10.1097/SLA.0000000000007191)
Supplement: Supplementary file 1 [file sla-284-0943-s001.docx]

Supplemental 1

Supplemental Table 1: Codes used for case selection in Statistics Netherlands registries

| Diagnosis codes | |
| --- | --- |
| ICD-9 code for rAAA | 441.3 |
| ICD-10 code for rAAA | I71.3 |
| Procedure codes | |
| CVV codes | 538330, 538331, 538332, 538333, 538334, 538335, 538336, 538337, 538338, 539520, 539525, 539575, 539590, 539595, 883653, 883656 |
| CBV codes | 033554, 033555, 033559, 033560, 033561, 033562, 080930, 333532, 333534, 333535, 333536, 333537, 380020, 385332, 333152X, 333153L, 333153X, 333153Y, 333154A, 333530A, 333530C, 333530G, 333530H, 333530J, 333530K, 330530M, 330530N, 330530P, 330530Q, 330530R, 330532F, 330533A, 330536D, 330536E, 388932A |
| ZA codes | 033554, 033555, 033559, 033560, 033561, 033562, 080930 |

Supplemental Table 2: Baseline characteristics for the included cohort

|  | EVAR  (n=1616) | Open Repair  (n = 1983) | P-value | Missing (%) |
| --- | --- | --- | --- | --- |
| % | 44.9%% | 55.1% | <0.001 | 0.0 |
| Sex (% male) | 86.9% (1405) | 82.9% (1647) | 0.001 | 0.0 |
| Age (mean) (SD)  (median) (IQR) | 75.1 (8.5)  75.3 (69.2, 81.7) | 73.7 (7.77)  74.2 (68.8, 79.2) | <0.001 | 0.0 |
| Aneurysm size (mm) (IQR) | 76.0 (65.0, 90.0) | 80.0 (69.0, 91.0) | <0.001 | 5.9 |
| Hemoglobin (mmol/L) (IQR) | 7.3 (6.2, 8.2) | 7.4 (6.4, 8.2) | <0.001 | 3.7 |
| Systolic blood pressure mmHg (IQR) | 110 (87, 134) | 105 (80, 130) | 0.407 | 6.8 |
| Glasgow Coma Scale (%) |  |  | 0.002 | 17.4 |
| 15 | 79.7% (1075) | 76.3% (1236) |  |  |
| 12-14 | 14.8% (200) | 14.9% (241) |  |  |
| 9-11 | 2.6% (35) | 3.2% (52) |  |  |
| <9 | 2.9% (39) | 5.6% (91) |  |  |
| AAA Type (%) |  |  | <0.001 | 17.1 |
| Infrarenal | 97.9% (1370) | 69.9 (1103) |  |  |
| Female (%) | 13.2% (181) | 16.6% (183) |  |  |
| Juxtarenal | 1.7% (24) | 28.2% (445) |  |  |
| Female (%) | 8.3% (2) | 20.0% (89) |  |  |
| Suprarenal | 0.4% (5) | 2.0% (31) |  |  |
| Female (%) | 40.0% (2) | 29.0% (9) |  |  |
| Earlier AAA repair (%) | 10.3% (144) | 5.1% (81) | <0.001 | 16.5 |
| Charlson Comorbidity Index (%) |  |  | <0.572 | 16.2 |
| 0 | 0.3% (4) | 0.5% (8) |  |  |
| 1 | 43.1% (579) | 44.8% (750) |  |  |
| 2 | 30.5% (410) | 30.4% (509) |  |  |
| >3 | 26.1% (350) | 24.3% (407) |  |  |
| Length of Stay (days) (IQR) | 6.0 (4.0, 11.0) | 10.0 (3.0, 19.0) | <0.001 | 0.0 |
| Length of Stay on ICU (days) (SD) | 3.8 (7.0) | 7.2 (9.9) | <0.001 | 39.4 |
| Prior Homecare (%) | 10.3% (155) | 6.4% (115) | <0.001 | 8.6 |
| Admission from institutional care (%) | 3.8% (61) | 3.0% (59) | 0.218 | 0.0 |

Supplemental Table 3: Ninety-day all-cause mortality following RAAA repair, stratified by sex, age group and repair type

| 90-day mortality | Male | Female | P-value |
| --- | --- | --- | --- |
| Overall | 29.2% (891) | 39.1% (214) | <0.001 |
| EVAR | 20.5% (288) | 26.5% (56) | 0.048 |
| < 75 years | 12.2% (87) | 21.0% (17) |  |
| > 75 years | 29.0% (201) | 30.0% (39) |  |
| Open repair | 36.6% (603) | 47.0% (158) | <0.001 |
| < 75 years | 29.2% (280) | 32.8% (44) |  |
| > 75 years | 46.9% (323) | 56.4 % (114) |  |

Supplemental Table 4: Multivariable logistic regression for 90-day mortality

| Predictor | Adjusted OR (95% CI) | P-value |
| --- | --- | --- |
| Female sex | 1.286 (1.053-1.567) | 0.013 |
| Age (per year) | 1.070 (1.060-1.082) | <0.001 |
| Open repair | 2.658 (2.274-3.113) | <0.001 |

Supplemental Table 5: Extended multivariable logistic regression for 90-day mortality

| Predictor | Adjusted OR (95% CI) | P-value |
| --- | --- | --- |
| Female sex | 1.125 (0.871-1.448) | 0.364 |
| Age (per year) | 1.068 (1.055-1.082) | <0.001 |
| Open repair | 2.540 (2.093-3.090) | <0.001 |
| AAA size (per mm) | 0.998 (0.993-1.004) | 0.516 |
| Hb deviation (per mmol/L)^1^ | 0.951 (0.885-1.021) | 0.162 |
| Systolic blood pressure (per 10 mmHg) | 0.936 (0.909-0.963) | <0.001 |
| Charlson Comorbidity Index (per point) | 1.258 (1.194-1.326) | <0.001 |
| Glasgow Coma Scale 12-14 | 1.684 (1.308-2.163) | <0.001 |
| Glasgow Coma Scale 9-11 | 3.232 (1.920-5.492) | <0.001 |
| Glasgow Coma Scale <9 | 3.850 (2.416-6.195) | <0.001 |

^1^Hb deviation was used instead of raw Hb to remove physiological sex-related differences in baseline hemoglobin, so that the variable reflects deviation from expected Hb for sex rather than sex itself.

Supplemental Table 6: Extended multivariable logistic regression for 90-day mortality for infrarenal RAAA’s

| Predictor | Adjusted OR (95% CI) | P-value |
| --- | --- | --- |
| Female sex | 0.956 (0.685-1.326) | 0.791 |
| Age (per year) | 1.072 (1.055-1.089) | <0.001 |
| Open repair | 2.765 (2.184-3.511) | <0.001 |
| AAA size (per mm) | 1.001 (0.994-1.007) | 0.838 |
| Hb deviation (per mmol/L)^1^ | 0.924 (0.846-1.009) | 0.077 |
| Systolic blood pressure (per 10 mmHg) | 0.936 (0.902-0.970) | <0.001 |
| Charlson Comorbidity Index (per point) | 1.285 (1.204-1.372) | <0.001 |
| Glasgow Coma Scale 12-14 | 1.951 (1.436-2.645) | <0.001 |
| Glasgow Coma Scale 9-11 | 3.705 (1.821-7.621) | <0.001 |
| Glasgow Coma Scale <9 | 4.021 (2.216-7.410) | <0.001 |

^1^Hb deviation was used instead of raw Hb to remove physiological sex-related differences in baseline hemoglobin, so that the variable reflects deviation from expected Hb for sex rather than sex itself.

Supplemental Table 7: Observed survival in RAAA population (%), in the matched general Dutch population (%), and the relative survival (Observed/Expected).

|  | Male | | | Female | | | |
| --- | --- | --- | --- | --- | --- | --- | --- |
| *Under 75* | **Observed** | **Expected** | **Relative** | | **Observed** | **Expected** | **Relative** |
| 1-year | 95.9 | 98.4 | 97.5 (96.4-98.6) | | 95.5 | 98.9 | 96.5 (93.3-99.9) |
| 3-year | 89.3 | 94.9 | 94.1 (92.3-95.9) | | 88.8 | 96.3 | 92.3 (87.1-97.8) |
| 5-year | 81.4 | 90.7 | 89.7 (87.2-92.4) | | 78.7 | 93.2 | 84.4 (76.7-92.9) |
| *Above 75* |  |  |  | |  |  |  |
| 1-year | 90.1 | 93.5 | 96.5 (94.3-98.5) | | 88.9 | 95.2 | 93.3 (88.5-98.2) |
| 3-year | 70.1 | 80.1 | 87.6 (83.6-91.6) | | 74.3 | 84.8 | 87.6 (80.1-95.7) |
| 5-year | 52.0 | 66.5 | 78.3 (72.7-84.2) | | 49.8 | 73.2 | 68.0 (57.1-80.9) |

| Readmissions in first 90 days | Male < 75 | Male > 75 | Female < 75 | Female > 75 | P-value |
| --- | --- | --- | --- | --- | --- |
| Proportion of patients with readmission | 16.0% | 16.4% | 17.7% | 16.0% | 0.824 |
| Mean number of readmissions (SD) | 1.4 (0.68) | 1.54 (0.95) | 1.39 (0.68) | 1.24 (0.43) | 0.534 |
| Days to first readmission (IQR) | 15.0 (5.5-42.5) | 16.0 (6.0-39.9) | 17.5 (7.3-25.8) | 15.5 (5.3-34.0) | 0.880 |
| Length of stay (SD) | 6.0 (8.4) | 6.0 (7.3) | 5.9 (9.3) | 6.0 (6.5) | 0.186 |
| Readmission cause |  |  |  |  |  |
| Cardiovascular | 19.7% | 20.5% | 18.7% | 21.5% | 0.806 |
| Complication | 20.9% | 17.0% | 13.1% | 16.3% | 0.004 |

Supplemental Table 8: All cause readmissions in the first 90 days

Supplemental Figure 1: Age-specific mortality following acute repair for periods 1995-2004^1^ and 2014-2023

^1^For both periods shown, mortality estimates were derived from Statistics Netherlands (SN) data to ensure comparability. Patients were identified using diagnostic codes for ruptured abdominal aortic aneurysm (ICD-9 code 441.3 for earlier years and ICD-10 code I71.3 for later years) combined with procedural codes indicating surgical repair of RAAA. Note that this approach captures all SN-registered surgically treated RAAA cases and may therefore yield slightly different mortality estimates compared with the DSAA-based cohort used in the primary analysis. Over time, the absolute number of repairs declined despite substantial population growth among individuals aged ≥60 years (+44% in women and +68% in men). After normalization for population size, RAAA repair incidence decreased from 346 to 146 per 100,000 in men and from 45 to 21 per 100,000 in women between the periods, while the turndown rate decreased in the same time periods.

Supplemental Figure 2: Annual proportion of EVAR procedures in the studied period by sex

Supplemental Figure 3: Kaplan-Meier survival curve for 90-day survivors by sex and age-groups

Supplemental Figure 4: Alluvial diagram for destination after discharge and homecare for men and women aged under 75

Supplemental Figure 5: Alluvial diagram for destination after discharge and homecare for men and women aged over 75

Supplemental Figure 6: Alluvial diagram for destination after discharge and homecare following EVAR or OR

Supplemental Figure 7: Cumulative incidence of reinterventions after repair estimated using Aalen-Johansen
