# Supplementary material for: Sex- and Age-specific Outcomes After Ruptured Abdominal Aortic Aneurysm Repair: A Nationwide Cohort Study
Source: Ann Surg. 2026 Aug 14;284(5):943–9. doi: 10.1097/SLA.0000000000007191 (PMC13652115; doi:10.1097/SLA.0000000000007191)
Supplement: Supplementary file 2 [file sla-284-0943-s002.docx]

Table 9: Baseline characteristics by sex

|  | Male | Female | P-value | Missing (%) |
| --- | --- | --- | --- | --- |
| N | 84.8% (3090) | 15.2% (552) | <0.001 | 0.0 |
| Age (mean) (SD)  (median) (IQR) | 74.0 (8.2)  74.2 (68.7, 79.9) | 76.3 (7.9)  77.2 (71.6, 81.9) | <0.001 | 0.0 |
| Aneurysm size (mm) (IQR) | 80.0 (68.0, 91.0) | 70.6 (60.0, 80.0) | <0.001 | 5.9 |
| Hemoglobin (mmol/L) (IQR) | 7.4 (6.4, 8.3) | 6.8 (5.8, 7.7) | <0.001 | 3.7 |
| Systolic blood pressure mmHg (IQR) | 108 (84, 133) | 107 (83, 130) | 0.407 | 6.8 |
| Glasgow Coma Scale (%) |  |  | 0.023 | 17.4 |
| 15 | 78.7% (2010) | 73.5% (333) |  |  |
| 12-14 | 14.3% (366) | 17.7% (80) |  |  |
| 9-11 | 2.6% (67) | 4.6% (21) |  |  |
| <9 | 4.4% (112) | 4.2% (19) |  |  |
| Repair Type (% open) | 54.0% (1647) | 61.4 (336) | 0.002 | 1.2 |
| AAA Type (%) |  |  | 0.003 | 17.1 |
| Infrarenal | 84.0% (2141) | 78.3% (396) |  |  |
| *OR (%)* | *42.9% (920)* | *46.2% (183)* |  |  |
| Juxtarenal | 15.0% (383) | 19.3% (91) |  |  |
| *OR (%)* | *93.0% (356)* | *97.8% (89)* |  |  |
| Suprarenal | 1.0% (26) | 2.3% (11) |  |  |
| *OR (%)* | *84.6% (22)* | *81.2% (9)* |  |  |
| Earlier AAA repair (%) | 8.2% (211) | 5.7% (27) | 0.080 | 16.5 |
| Charlson Comorbidity Index (%) |  |  | 0.274 | 16.2 |
| 0 | 0.5% (13) | 0.0% (0) |  |  |
| 1 | 44.2% (1139) | 42.9% (203) |  |  |
| 2 | 30.6% (789) | 29.6% (140) |  |  |
| >3 | 24.7% (637) | 27.5% (130) |  |  |
| Prescribed medication^1^ (%) |  |  |  |  |
| Statins | 52.2% (1612) | 52.7% (291) | 0.848 | 0.0 |
| Anti-hypertensives | 62.6% (1935) | 69.0% (381) | 0.005 | 0.0 |
| Antiplatelets | 52.3% (1616) | 51.6% (285) | 0.808 | 0.0 |
| Length of Stay on ICU (days) (IQR) | 2.0 (2.0, 5.0) | 3.0 (2.0, 6.0) | 0.197 | 39.4 |
| Length of Stay (days) (IQR) | 8.0 (4.0, 15.0) | 9.0 (3.0, 17.0) | 0.428 | 0.0 |
| Prior Homecare (%) | 6.9% (195) | 15.2% (78) | <0.001 | 8.6 |
| Admission from institutional care (%) | 3.1% (95) | 4.9% (27) | 0.040 | 0.0 |

**Figure 7: Relative survival following successful repair compared to the Dutch general population**

Figure 8: Alluvial diagram for destination after discharge and homecare for men and women.

Note: Home care was analysed exclusively for patients who returned home following hospital discharge. Although patients may subsequently return home after stays in nursing homes or rehabilitation centres, these pathways have not been visualized to maintain clarity in the diagram.
